# Green material: ecological importance of imperative and sensitive chemi-sensor based on Ag/Ag_2_O_3_/ZnO composite nanorods

**DOI:** 10.1186/1556-276X-8-380

**Published:** 2013-09-08

**Authors:** Abdullah M Asiri, Sher Bahadar Khan, Mohammed M Rahman, Abdullah G Al-Sehemi, Saleh A Al-Sayari, Mohammad Sultan Al-Assiri

**Affiliations:** 1 Center of Excellence for Advanced Materials Research (CEAMR), King Abdulaziz University, P.O. Box 80203, Jeddah 21589, Saudi Arabia; 2 Chemistry Department, Faculty of Science, King Abdulaziz University, P.O. Box 80203, Jeddah 21589, Saudi Arabia; 3 Department of Chemistry, Faculty of Science, King Khalid University, P.O. Box 9004, Abha 61413, Saudi Arabia; 4 Advanced Materials and NanoResearch Centre, Najran University, P.O. Box 1988, Najran 11001, Saudi Arabia; 5 Department of Physics, College of Science and Arts, Najran University, P.O. Box 1988, Najran 11001, Saudi Arabia

**Keywords:** Composite nanorods, Structural properties, Optical properties, Phenyl hydrazine sensing

## Abstract

In this report, we illustrate a simple, easy, and low-temperature growth of Ag/Ag_2_O_3_/ZnO composite nanorods with high purity and crystallinity. The composite nanorods were structurally characterized by field emission scanning electron microscopy, X-ray powder diffraction, Fourier transform infrared spectroscopy, and X-ray photoelectron spectroscopy which confirmed that synthesized product have rod-like morphology having an average cross section of approximately 300 nm. Nanorods are made of silver, silver oxide, and zinc oxide and are optically active having absorption band at 375 nm. The composite nanorods exhibited high sensitivity (1.5823 μA.cm^−2^.mM^−1^) and lower limit of detection (0.5 μM) when applied for the recognition of phenyl hydrazine utilizing I-V technique. Thus, Ag/Ag_2_O_3_/ZnO composite nanorods can be utilized as a redox mediator for the development of highly proficient phenyl hydrazine sensor.

## Background

Metal oxide-based nanomaterials are of growing interest owing to their inimitable properties, distinctive performance, and extensive relevance in various fields especially in sensor technology which is a forefront technology because of its prominent role in environmental, industrial, medicinal, and clinical monitoring [1-3]. The extensive applications of nanomaterials as sensing materials are generally considered due to their small size, particular shape, high active surface-to-volume ratio, and high surface activity. These properties make nanomaterials attractive in many fields and especially in sensor technology [4-6]. The small particle size and active surface area of nanomaterial make them capable to detect and investigate sensing analytes in very low concentration, and therefore, nanomaterials are capable to detect and monitor the toxic chemicals and organic pollutants in the environment at very low concentration which is impossible for a sensor with microstructure materials. Therefore, nanomaterials have created a center of interest for their use in chemical sensor fabrication [7,8].

Zinc oxide (ZnO) (wurtzite structure and large bandgap (3.37 eV) and high exciton binding energy (60 meV)) has been explored for various applications such as fabricating solar cells, sensors, catalysts, etc. ZnO has shown electrical, optical, and sensing properties which are largely dependent on the structural behaviors of ZnO that normally change due to the intrinsic defects which exist in ZnO and cause divergence of ZnO from the stoichiometry [9-11]. However, to expand the applications of ZnO to convene the rising desires for different purposes, there is a need to modify the features of ZnO. Doping of nanomaterials by adding dopant is a well-known and momentous method to alter the features of the nanomaterials. Doped nanomaterials have recently shown excellent properties in various sectors. Doping process increases the surface area and trims down the size of nanomaterials and, as a result, enhances physical and chemical performance of nanomaterials [12-15].

Nowadays, the world is facing environmental pollution problem, and industrial development is mainly responsible for this environmental issue [1-4]. The industrial development is only beneficial if there is intelligent monitoring and proper control of the pollutant discharge to the environment as result of industrial process. These industries discharge various pollutants in gas and liquid form to the environment which are responsible for the environmental pollution [5-7]. One of these pollutants is waste liquid which causes contamination, eutrophication, and perturbation in aquatic life. Waste liquid discharges various organic pollutants to the environment such as hydrazine derivatives, liquid ammonia, dyes, phenols, etc. Hydrazine and its derivatives such phenyl hydrazine are well-known organic pollutant and industrial chemicals which discharge to the environment from their uses in industries and as aerospace fuels [16,17]. It is one of the great challenges to control these pollutants in the environment and protect the human and aquatic life.

Various techniques and materials have been used to develop susceptible and consistent analytical technique to monitor and protect the environment from toxic nature of phenyl hydrazine. Among these techniques, electro-analytical method using various redox mediators has proven itself as one of the simple and well-organized technique for the recognition of various pollutants [10-12]. Here, we proposed ZnO composite nanorods as a sensor material for the detection of phenyl hydrazine by electrochemical method to overcome the lower over potential of the conventional electrode and show good performance in terms of sensitivity by improving electrochemical oxidations. Metal oxide nanostructures have been used as a redox mediator to overcome the lower over potential of the conventional electrodes used in electro-analytical method and have shown good performance in terms of sensitivity by improving electrochemical oxidations [1-3]. Several reports in literature are related to pure and doped nanomaterials, but there is no literature about electrochemical properties of composite nanomaterials for phenyl hydrazine detection in aqueous phase. To get the utmost profit of the assets of nanomaterial, several methods have been established. However, we have used simple, low-cost, and low-temperature hydrothermal method for the synthesis of composite nanorods.

The aim of this involvement was to prepare, characterize, and investigate chemical sensing performance of composite nanorods based on Ag/Ag_2_O_3_/ZnO. The morphological, structural, and optical properties of the prepared nanorods were characterized by field emission scanning electron microscopy (FESEM), X-ray powder diffraction (XRD), Fourier transform infrared spectroscopy (FT-IR), X-ray photoelectron spectroscopy (XPS), and ultraviolet–visible (UV–vis) spectroscopy. Chemical sensing property was studied by simple I-V technique and detected phenyl hydrazine in aqueous solution with high sensitivity and selectivity.

## Methods

### Materials and methods

Silver chloride, zinc chloride, ammonium hydroxide, and all other chemicals are purchased from Aldrich Chemical Co (Milwaukee, WI, USA). All the chemicals are of reagent grade and used without further purification. Distilled water is used throughout the study. Composite nanorods were prepared by simple hydrothermal method. Then, 0.1 M aqueous solution of AgCl_2_ and ZnCl_2_ was prepared and then, the solution was made basic (pH = 10.0) by adding NH_4_OH solution. The basic solution was heated up to 150°C for 12 h in Teflon-lined autoclave. After stopping the reaction, the solvent was poured out and the precipitate is washed several times. Composite nanorods are acquired after drying the precipitate at room temperature and then calcined at 400°C for 5 h.

### Possible growth mechanism of ZnO

**Figure 1:**
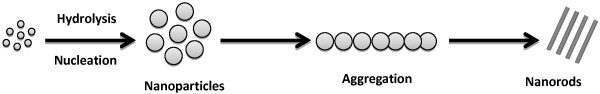
Possible growth mechanism of composite nanorods.

Initially, ZnCl_2_ and AgCl_2_ undergo hydrolysis in water in the presence of NH_4_OH and produce Zn^+^, Ag^+^, and OH^−^ which later produce Zn(OH)_2_ and Ag(OH)_2_. The heating cause the dehydration of Zn(OH)_2_ to ZnO and Ag_2_O_3_. During growth process (Figure 1), first ZnO and Ag_2_O_3_ nucleus growth takes place which then aggregate and produce Ag/Ag_2_O_3_/ZnO nanoparticles by Ostwald ripening. The nanoparticle crystallizes and aggregates with each other through Van der Waals forces and hydrogen bonding and gives Ag/Ag_2_O_3_/ZnO composite nanorods.

### Fabrication of sensor

Gold electrode was fabricated with composite nanorods using butyl carbitol acetate and ethyl acetate as a conducting coating binder. Then, it was kept in the oven at 60°C for 3 h until the film is completely dried. Next, 0.1 M phosphate buffer solution at pH 7.0 was made by mixing 0.2 M Na_2_HPO_4_ and 0.2 M NaH_2_PO_4_ solution in 100.0 mL de-ionize water. A cell was constructed consisting of composite nanorods coated with AuE as a working electrode, and Pd wire was used as a counter electrode. Phenyl hydrazine solution was diluted at different concentrations in DI water and used as a target chemical. The amount of 0.1 M phosphate buffer solution was kept constant as 10.0 mL during the measurements. The solution was prepared with various concentration ranges of target compound (1.7 mM to 17.0 M). The ratio of voltage and current (slope of calibration curve) is used as a measure of phenyl hydrazine sensitivity. Detection limit was calculated from the ratio of 3 N/S (ratio of noise × 3 vs. S) versus sensitivity in the linear dynamic range of calibration plot. Electrometer is used as a voltage sources for I-V measurement in a simple two-electrode system.

### Characterization

X-ray diffraction patterns (XRD) were taken with a computer-controlled X’Pert Explorer, PANalytical diffractometer (PANalytical, Almelo, The Netherlands). X-ray diffractometer was operated at 40 kV/20 mA in continuous scan mode at a scanning speed of 0.02° (2*θ*s)^−1^ with a slit of 1°. The surface morphology of composite nanorods was studied at 15 kV using a JEOL scanning electron microscope (JSM-7600 F, JEOL Ltd., Akishima-shi, Japan). FT-IR spectra was recorded in the range of 400 to 4,000 cm^−1^ on PerkinElmer (spectrum 100, Waltham, MA, USA) FT-IR spectrometer. UV spectra was recorded from 250 to 800 nm using PerkinElmer (Lambda 950) UV–vis spectrometer.

## Results and discussion

### Structural and morphological characterization

**Figure 2:**
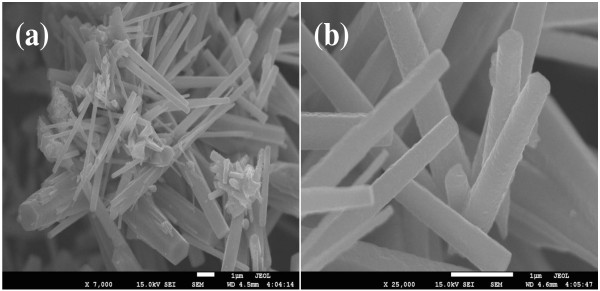
Typical (a) low-magnification and (b) high-resolution FESEM images of composite nanorods.

The morphology of the synthesized product was characterized by FESEM which is shown in Figure 2a,b. Low and high magnifications of FESEM images demonstrate that the composite material has rod-shape morphology with average cross section of approximately 300 nm. The nanorods are grown in high density.

**Figure 3:**
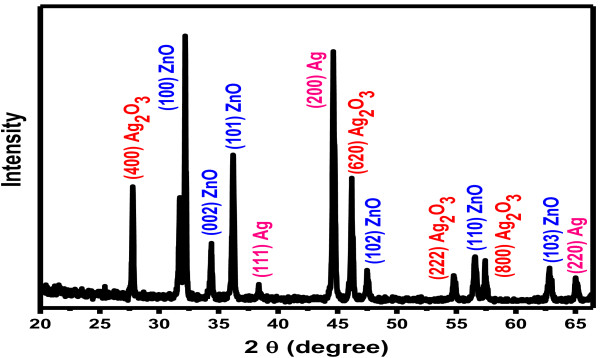
Typical XRD pattern of composite nanorods.

The crystallinity of composite nanorods was studied by X-ray powder diffraction, and the results are illustrated in Figure 3. XRD spectrum of the nanorods exhibited diffraction peaks associated to Ag (JCPDS # 04–0783), Ag_2_O_3_ (JCPDS # 40–909), and ZnO (JCPDS # 36–1451) with wurtzite hexagonal phase. All the attributed peaks are suited with Ag, Ag_2_O_3_, and ZnO. There is no additional impurity peak in X-ray diffraction spectrum which indicates that the prepared nanorods are well-crystalline composite of Ag, Ag_2_O_3_, and ZnO.

**Figure 4:**
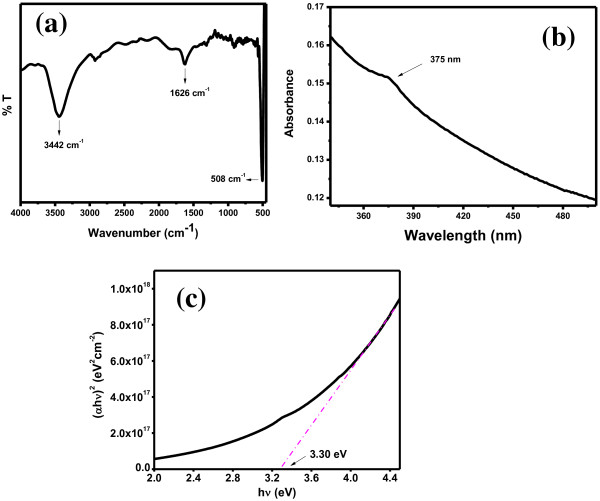
**Typical FT-IR and UV–vis spectra of composite nanorods. (a)** Chemical structure, **(b)** optical property, and **(c)** bandgap energy *E*_g_ of composite nanorods.

The chemical structure of composite nanorods was evaluated by FT-IR spectroscopy, shown in Figure 4a. FT-IR spectrum of composite nanorods is measured in the range of 400 to 4,000 cm^−1^ and shown in Figure 4a. FT-IR spectrum showed absorption at 508, 1,626, and 3,442 cm^−1^. The band centered at 3,442 cm^−1^ (O-H stretching) and 1,626 cm^−1^ (O-H bending) is attributed to moister absorbed [1,7]. The very intense and broad band centered at 508 cm^−1^ is responsible for M-O (M = Zn and Ag) bonds [9-12].

The optical property of the composite nanorods is important assets which was studied using a UV–vis spectrophotometer and shown in Figure 4b. UV–vis absorption spectrum displayed absorption peak at 375 nm without other impurity peak. The bandgap energy *E*_g_ of composite nanorods was found to be around 3.30 eV from the tangent drawn at linear plateau of curve (α*hν*)^*2*^ vs. *hν* (Figure 4c).

**Figure 5:**
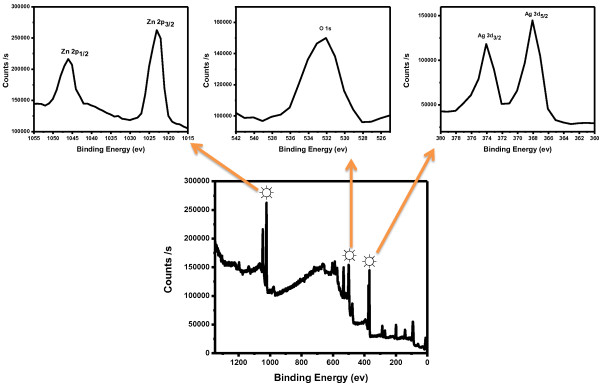
XPS spectrum of composite nanorods.

Figure 5 shows XPS spectrum of composite nanorods which gives information about the bonding configuration and composition of the synthesized nanorods. XPS spectrum of composite nanorods displayed photoelectron peaks for Ag 3d_5/2_, Ag 3d_3/2_, O 1 s, Zn 2p_3/2_, and Zn 2p_1/2_ at binding energies of 368.0, 374.0, 532.2, 1,023.1, and 1,046.1 eV, respectively, which specifies that composite nanorods contain oxygen, zinc, and silver. These results are similar to the reported values in literature [18,19]. The XPS data reflect that composite nanorods are made of Ag, Ag_2_O_3_, and ZnO.

### Chemical sensing properties

**Figure 6:**
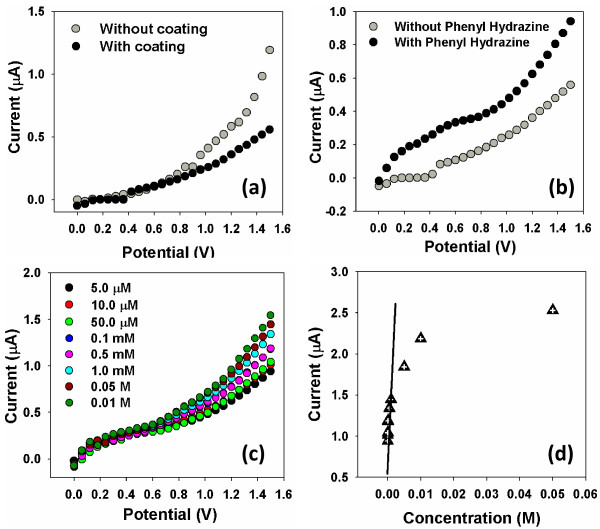
**I-V characterization of composite nanorods. (a)** Current comparison of composite nanorods coated and un-coated Au, **(b)** comparison of coated electrode current with and without phenyl hydrazine, **(c)** concentration variation of phenyl hydrazine, and **(d)** calibration plot.

Composite nanorods were employed for finding phenyl hydrazine by measuring the electrical response of phenyl hydrazine using I-V technique [1-3]. The electrical current of bare gold electrode and nanorod-layered gold electrode (working electrode) is shown in Figure 6a. Figure 6b shows current of working electrode without phenyl hydrazine and with 100.0 μL phenyl hydrazine. It is obvious that the addition of phenyl hydrazine enhances electrical current which suggests that composite nanorods are sensitive to phenyl hydrazine. Thus by insertion of phenyl hydrazine, augmentation in electrical current implies that nanorods has fast and susceptible response to the phenyl hydrazine. The rapid electron swap and good electro-catalytic oxidation properties are accountable for the high electrical response of composite nanorods to phenyl hydrazine [7-9].

Phenyl hydrazines easily undergo catalytic dissociation reaction by applying to I-V technique and generate diazenyl benzene, 2H^+^, and 2e^–^ which cause increase in electrical conductivity [10,11].

Generally, electron emission takes place from the chemisorbed oxygen into the conduction band of the sensor and ionizes atmospheric oxygen molecules by giving electron from the conduction band and ionosorbed on the surface as O_ads_^−^ (O^−^ or O_2_^−^ depending on the energy available). The resulting equation is

(1) $$O_{2}+{2e}^{-}\to {2O}_{\mathrm{ads}}{}^{-}$$

**Figure 7:**
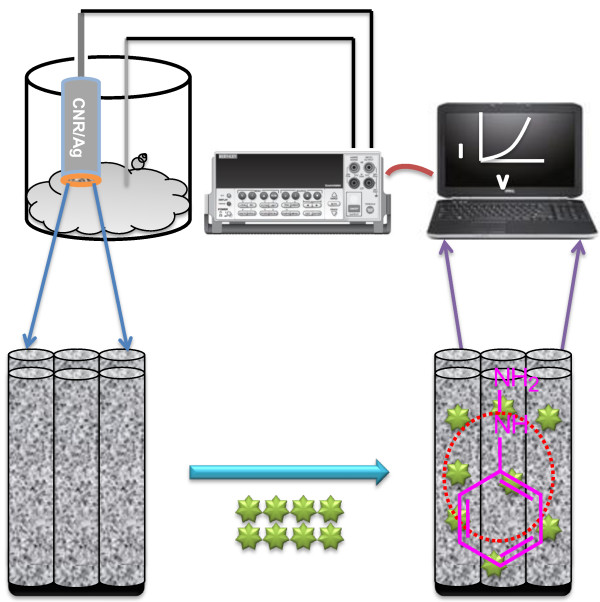
Mechanism of phenyl hydrazine in the presence of composite nanorods.

The surface adsorbed oxygen (O_ads_^−^) reacts with diazenyl benzene produced by the catalytic reaction of phenyl hydrazine and produce benzenediazonium ion (Figure 7) [12-15].

The electrical response of phenyl hydrazine was studied in the concentration assortment of 5.0 μM to 0.01 M by consecutive addition into 0.1 M PBS solution with constant stirring, and the outcomes are given away in Figure 6c. The results show increase in electrical current is directly proportional to the concentration of phenyl hydrazine which increased with increase in concentration of phenyl hydrazine. The gradual increase in current suggests that the number of ions increases with increase in phenyl hydrazine concentration by giving extra electron to the conduction band of composite nanorods [16,17].

The calibration curve was plot out from the current variation and is depicted in Figure 6d. The calibration curve indicates that at first, current raises with rise in phenyl hydrazine concentration but behind definite concentration, the current turns into constant which reflects saturation at this specific concentration. The lower part of the calibration curve is linear with correlation coefficient (*R*) of 0.8942, while the slope of this linear lower part gave sensitivity which is 1.5823 μA.cm^−2^.μM^−1^. Composite nanorods displayed linear dynamic range from 5.0 μM to 1.0 mM and detection limit of 0.5 μM. The linear part of composite nanorods is the receptive region for phenyl hydrazine which indicates that it is very sensitive and will detect phenyl hydrazine at trace level. The developed sensors would be useful at lower phenyl hydrazine concentration [10-14].

By comparing with reported literature, composite nanorod-based phenyl hydrazine sensor was found to be more sensitive (Table 1). Composite nanorods illustrated drastically elevated sensitivity and lower detection limit as compared to earlier reported phenyl hydrazine sensors [17,20,21]. Consequently, the composite nanorods are excellent aspirant for the development of competent and most sensitive phenyl hydrazine sensor.

**Table 1:** Comparison between the sensitivity of composite nanorod sensor and literature

**Electrode materials**	**Sensitivity (μA.cm**^**−2**^**.μM**^**−1**^**)**	**Reference**
Composite nanorods	1.5823	Present work
Al/ZnO	1.143	[17]
Carbon nanotube	0.03	[20]
Ferrocene and carbon nanotubes	0.0389	[21]

## Conclusions

In summary, composite nanorods were synthesized by a simple and low-temperature hydrothermal process. The detailed morphology of the synthesized composite nanorods was characterized by XRD, FESEM, FT-IR, XPS, and UV–vis spectra and reveals that the synthesized composite is well-crystalline optically active nanorods containing Ag, Ag_2_O_3_, and ZnO. The synthesized composite nanorods were applied for the detection and quantification of phenyl hydrazine in liquid phase. The performance of the developed phenyl hydrazine sensor was excellent in terms of sensitivity, detection limit, linear dynamic ranges, and response time. Since synthesized composite nanorods have very simple synthetic procedure, low cost, and high sensitivity for phenyl hydrazine sensing, therefore, it is concluded that chemical sensing properties of composite nanorods are of great importance for the application of composite nanorods as a chemical sensor.

## Competing interests

The authors declare that they have no competing interests.

## Authors’ contributions

AMA, SBK, and AGAS carried out the synthesis and characterization of composite nanorods. MMR carried out the sensing study of nanorods. MSAA and SAAS provided all the instruments used for characterization and helped in characterization of the nanomaterial. All authors read and approved the final manuscript.
